# State-of-the-Art Nanomaterials in Energy and Environmental Applications

**DOI:** 10.3390/ma19010019

**Published:** 2025-12-20

**Authors:** Sivaprakash Paramasivam, Govindasamy Palanisamy

**Affiliations:** 1 Department of Mechanical Engineering, Keimyung University, Daegu 42601, Republic of Korea; 2 School of Chemical Engineering, Yeungnam University, 280 Daehak-Ro, Gyeongsan 38541, Republic of Korea

## 1. Introduction and Scope

Advanced nanosized materials are typically used to facilitate developments in electronics, batteries, supercapacitors, the medical field, environmental remediation [1,2,3,4,5], etc. Nanomaterials are smaller than 100 nm, with a high surface area and tunable size and shape. These distinct properties make them well suited to a variety of applications, such as energy storage, wastewater treatment, drug delivery systems, engineering living materials, biomedical applications, and biofilms [6,7,8,9,10]. As Guest Editors of this Special Issue, our primary focus is on the design of nanostructures and their use in energy and environmental applications.

As of late, the use of nanomaterials in supercapacitor and battery applications has received greater attention due to their long cycle stability, impressive power delivery, and increased storage capacity [11]. When combined in a hybrid formation with polymers or other zero- and two-dimensional materials, these nanomaterials can form a strong, conductive interface with the electrolytes [12,13]. This behavior improves the performance of portable electronics and vehicles by affording them qualities such as a high specific capacitance, longer cycle life, etc. Alternatively, hybrid nano systems are also widely used in adsorbents and catalysts for the removal of hazardous dyes and contaminants [14]. Moreover, the widespread pursuit of green approaches has seen the development of eco-friendly nanomaterials and green synthesis materials for use in water treatment technologies, which have been shown to be effective as well as environmentally safe and sustainable [15]. This Special Issue also features investigations into engineering living materials (ELMs), materials that can sense their surroundings, adapt, self-heal, and perform useful biological functions, showing great potential for application in the areas of energy production, bioremediation, and medicine [16].

This Special Issue focuses on nanostructured materials designed for application in supercapacitors, wastewater treatment, tissue scaffolds, drug delivery, and environmental toxicology. Its aim is to highlight clear, scalable, and new solutions in the field that can be understood and applied by researchers. By presenting this collection detailing the recent advances in nanostructures and related materials, this Issue hopes to encourage the development of practical technologies that will help meet the growing global demand for energy and environmental applications.

## 2. Contributions

In the work of [17], nitrogen (N)- and sulfur (S)-doped carbon dots (NS-CDs) were synthesized from p-phenylenediamine and thioacetamide. The NS-CDs were chosen as the fluorescent sensing platform because the incorporation of N and S heteroatoms enriches surface states, enhances electron distribution, and greatly increases photoluminescence efficiency, enabling excitation-independent fluorescence, strong photostability, good water dispersibility, and a quantum yield of 28%. These properties make NS-CDs highly advantageous for analytical applications, particularly for inner filter effect (IFE)-based fluorescence quenching systems. Quercetin (QT) is a polyphenolic flavonoid compound naturally found in fruits, vegetables, and beverages. QT is well known for its antiviral, anticarcinogenic, and anti-inflammatory activities as well as its ability to scavenge free radical species. However, excessive intake of QT may result in headache, kidney malfunction, and weak glutathione S-transferase activity. Hence, due to its biological importance, QT was selected as the target analyte. Interestingly, QT exhibits a strong absorption band near 370 nm that overlaps the excitation spectrum of NS-CDs at 375 nm, enabling the use of an IFE-based fluorescence sensing probe. The developed NS-CDs probe demonstrated excellent sensitivity and specificity for QT, with a limit of detection (LOD) of 17.3 nM and a linear range of 0–29.7 μM. The sensing probe also exhibited reliable performance even in the presence of structurally related amino acids, biomolecules, flavonoids, phenols, and metal ions. In real sample analysis, the NS-CDs probe performed well, accurately quantifying QT in onion extracts and red wine samples, with a recovery range of 93.87–102.27%. The observed results indicate that the NS-CDs probe can be employed as a facile, robust sensing system for monitoring QT in food samples. The proposed sensing probe can also be integrated into portable, rapid, on-site sensing platforms, such as smartphones.

The electrochemical work for the supercapacitor is covered in a later article [18], which discusses the recent emergence of metal oxides as a promising material for supercapacitor applications due to their pseudocapacitive properties. Though RuO_2_ has high pseudocapacitance, it is restricted by its cost and environmentally harmful nature. SnO_2_-based nanomaterials have also recently gained recognition as a good choice of electrode material for supercapacitor applications. However, their poor electrical conductivity and electrolyte ion transfer continue to limit their practical use. Hence, the doping or incorporation of nitrogen, metals, metal oxides, sulfides, carbon-based materials, and polymers has been used to further boost the electrochemical performance of metal oxides. Consequently, Ni, one of several divalent dopants, has the same radius as tetravalent Sn^2+^, with promising properties, including low cost, nontoxicity, chemical stability, and a highly efficient charge transfer process. Given this, the article discusses the application of Ni-doped SnO_2_ in supercapacitors, as well as the use of a hydrothermal technique for the preparation of materials. The powder XRD confirmed that the mixed phase of Ni/SnO_2_ has the tetragonal structure typical of SnO_2_ and the cubic structure of Ni in a crystalline nature. Interestingly, at various doping concentrations, Ni had a notable impact on the host lattice structure of SnO_2_. FTIR and EDAX analyses were used to confirm the elemental and functional groups in Ni/SnO_2_ nanomaterials, and the materials were subjected to electrochemical performance analysis. Furthermore, the CSP of the prepared nanomaterials with varying Ni dopant concentrations (1, 3, and 5%) in SnO_2_ was studied. A 5% Ni-doped SnO_2_ nanocomposite showed a maximum Csp of 841.85 F/g at 5 mV/s in an electrolyte of 6 M KOH. To improve electrochemical performance, a redox additive electrolyte was used, which had a maximum Csp of 2130.33 F/g at 5 mV/s and an excellent capacitance retention of 93.22% after 10,000 GCD cycles. An investigation into the effect of RAE (Redox Additive Electrolyte) reveals a capacitance that is roughly 2.5 times better than that of the KOH electrolyte. The KOH electrolyte and RAE both displayed faradaic pseudocapacitive properties, with this research article indicating this is expected to lead to new developments in high-performance supercapacitors.

The review article [19] demonstrates the ability of nanostructured silicon-based anode materials to significantly improve the electrochemical performance of Li-ion batteries by addressing challenges associated with Si, primarily its severe volume expansion during cycling. The designed shells improve structural integrity and electrochemical stability by minimizing continuous SEI (Solid Electrolyte Interphase) formation and reducing the direct electrolyte contact, reducing dramatic volume changes during the cycling process. Si@TiO_2_/Ag nanowires and Si@V_3_O_4_@C are examples of core–shell and double-shell nanostructures that efficiently buffer volume changes and improve electrical conductivity, leading to greater reversible capacities and better cycling stability. For instance, Si nanoparticles coated with TiO_2_ and combined with Ag nanowires (Si@TiO_2_/Ag) show synergistic advantages in mechanical robustness and electrical conductivity, with an initial discharge capacity of 3524 mAh g^−1^ at 400 mA g^−1^. The Si@NSC electrode can maintain 90.2% of its capacity (1720 mAh g^−1^) after 550 cycles at 0.3 A/g because N and S co-doped carbon shells produce defect-rich conductive networks that allow volume expansion. However, a single-shell structure design may fail under continuous cycles as a result of accumulated stress and shell fracture. To overcome this issue, multi-shell systems such as Si@V_3_O_4_@C provide enhanced structural stability and ion/electron transport. The inner layer of V_3_O_4_ offers rapid Li^+^ diffusion and mechanical stabilization, while the outer layer of carbon improves electrical conductivity and strain tolerance. Consequently, the Si@V_3_O_4_@C multi-shell structure achieves 1061.1 mAh g^−1^ after 700 cycles at 0.5 A g^−1^, with a Coulombic efficiency of 99.3%, and maintains 78.7% capacity retention in full cells paired with LiFePO_4_ after 130 cycles at 0.5 C. The results highlight the importance of rationally engineered core–shell designs in the development of high-performance, long-lasting silicon anodes.

The authors in [20] state that the widespread presence of formaldehyde in the environment makes the determination of its concentration a crucial objective. A simple, effective calorimetric method for detecting formaldehyde is presented, using Au nanoparticles functionalized with three amino-thiophenol isomers: 4-ATP (S1), 3-ATP (S2), and 2-ATP (S3). Au nanoparticles were synthesized using a citrate reduction process, conducted by boiling 100 mL of 1% HAuCl4 and reducing it with 2 mL of 1% trisodium citrate until a ruby-red colloid was formed. After adjusting the PH of the nanoparticle solution to 3, the AuNPs were functionalized without centrifugation by mixing them with ATP ligands in controlled ratios (9:1 for S1, 7:1 for S2, and 5:1 for S3). The thiol groups of ATPs formed strong Au-S bonds, allowing the amino groups to react with formaldehyde. UV-Vis spectroscopy confirmed successful functionalization by shifting the surface plasmon resonance peaks to 530 nm (S1), 535 nm (S2), and 527 nm (S3), as well as a visible color change from ruby red to violet red. Furthermore, DLS and SEM analysis revealed the initial particle size of the functionalized AuNPs to be 32.76 ± 1.6 nm for S1, 8.81 ± 1.12 nm for S2, and 3.91 ± 0.21 nm for S3, all confirming spherical and uniform morphology. The NPs strongly aggregated upon their reaction with formaldehyde, and their hydrodynamic diameters increased significantly (17-fold for S1, 10-fold for S2, and 20-fold for S3), suggesting a cross-linking mechanism brought on by the interaction between surface amines and aldehyde groups. FTIR spectra supported the mechanism through the elimination of the free thiol peaks at 2557/cm, confirming the Au-S bond, while N-H stretching bands were retained, confirming the presence of reactive amino groups. Furthermore, strong pH responsiveness was demonstrated by the sensors, which became more aggregated in acidic environments and more stable in alkaline ones as a result of OH- ion electrostatic shielding. Stability tests revealed that all sensors maintained nearly unchanged A650/A530 ratios for 96 h at RT and remained stable for up to 90 days at 40 °C, with S3 demonstrating the highest long-term stability and S1 the strongest formaldehyde responsiveness. In addition to this, an interference test confirmed that the use of ethanol, methanol, ethylene glycol, and toluene (each at 10 mM) revealed minimal changes in signal, confirming excellent selectivity. With the detection limit of 1.03 mM in ultrapure water and 1.5 mM in river water, quantitative titration experiments proved this calorimetric ATP-AuNP platform to be dependable, rapid, and appropriate for the real-world monitoring of formaldehyde in the environment.

The study of [21] investigates the assembly of deacetylated nano-whiskers into iridescent films with structural colors similar to those seen in natural organisms, focusing on the effects of ultrasonication, film thickness, and ionic strength on nanofiber alignment, multilayer development, and the optical appearance of the final films. The nanofibers, originally obtained as a viscous and highly entangled suspension, were subjected to controlled ultrasonification to shorten them and improve their dispersibility, effectively narrowing their width to about 3–5 nm and decreasing the average fiber length to about 133 nm. Additionally, this treatment reduced viscosity, resulting in favorable drying conditions for ordered self-assembly. After sonification, X-ray diffraction verified a slight decrease in crystallinity, suggesting the partial disruption of the chitin crystal domains. Furthermore, the nano-whiskers formed a multilayered structure, with periodic spacing between 270 and 330 nm when they were cast and dried, corresponding to visible light wavelengths and producing intense purple and green interference colors. Following this, AFM and FESEM cross-sectional analysis confirmed the distinct layered structure, minimal light scattering, and smooth film morphology, all of which contribute to the striking structural color. Film thickness was confirmed as a key factor in determining color strength; thicker films, which were formed from larger dispersion volumes, produced more layers, increasing internal reflections and scattering and reducing the color intensity, whereas thinner films showed intense coloration. The coffee-ring effect developed a thickness gradient that produced a concentric color pattern across the film. Additionally, the study showed that ionic strength had a significant impact on nano-whisker assembly. Higher concentrations (20–40 mM) were found to decrease surface charge, promote aggregation, and interfere with periodic layering, resulting in weak or absent coloration, whereas low concentrations (0–10 mM) maintained dispersion quality and vibrant colors. Overall, the article highlights that precise nanoscales can be achieved by regulating nanofiber dimensions, dispersion behaviors, film thickness, and ionic environment to achieve bright and tunable colors from chitin.

This Special Issue of Materials has attracted attention worldwide, and its success is evident in the publication of five papers of outstanding quality. The Guest Editors offer their sincere appreciation to all contributing authors and thank the reviewers for their time and valuable comments and suggestions. We are especially thankful to the Section Managing Editor and Ms. Hester Hu, whose dedication and continuous support played a key role in the completion of this issue. The Guest Editors also extend thanks to the Editor-in-Chief of *Materials* for providing us with the opportunity to manage this Special Issue. The supportive and collaborative cooperation of all of those mentioned above has contributed to the success of this Special Issue.

## References

[1] Słoma M. (2023). 3D printed electronics with nanomaterials. Nanoscale.

[2] Haleem A., Javaid M., Singh R.P., Rab S., Suman R. (2023). Applications of nanotechnology in medical field: A brief review. Glob. Health J..

[3] Poorshakoor E., Darab M. (2024). Advancements in the development of nanomaterials for lithium-ion batteries: A scientometric review. J. Energy Storage.

[4] Li J., Zhu J., Dong Z., Wu Q. (2023). Nanomaterials Derived from a Template Method for Supercapacitor Applications. ChemistrySelect.

[5] Khin M.M., Nair A.S., Babu V.J., Murugan R., Ramakrishna S. (2012). A review on nanomaterials for environmental remediation. Energy Environ. Sci..

[6] Kumar M., Chauhan H., Satpati B., Deka S. (2019). Yolk Type Asymmetric Ag-Cu_2_O Hybrid Nanoparticles on Graphene Substrate as Efficient Electrode Material for Hybrid Supercapacitors. Z. Fur Phys. Chem..

[7] Palanisamy G., Park S., Lee J.-H.J.H., Lee J.-H.J.H. (2023). Alizarin-loaded hydroxyapatite containing SnO_2_ nanoparticles for efficient dye removal and biofilm inhibitory properties. Appl. Surf. Sci..

[8] Velmurugan G., Ganapathi Raman R., Sivaprakash P., Viji A., Cho S.H., Kim I. (2023). Functionalization of Fluorine on the Surface of SnO_2_–Mg Nanocomposite as an Efficient Photocatalyst for Toxic Dye Degradation. Nanomaterials.

[9] Saeidirad M., Naderi N., Sehat S., Qazi F.S., Zare I., Imani M., Kang H., Mirshafiei M., Koohkhezri M., Sadat Z. (2025). Gelatin-integrated carbon nanomaterials in nanomedicine: From biosensing and bioprinting to tissue engineering and other biomedical applications. Adv. Drug Deliv. Rev..

[10] Ma J., Hai Y., Zheng K., Hu X., Ni K. (2025). Nanoparticle-based drug delivery systems in urologic oncology: From targeted therapy to precision theranostics. Mater. Today Bio.

[11] Mandal S., Mendhe A.B., Rakhade H.M., Barse N.S., Roy M., Rosaiah P., Park T., Lee H.S., Mendhe A.C., Kim D. (2025). Recent advancement and design in supercapacitor hybrid electrode materials: Bridging the gap between energy and power density. Chem. Eng. J. Adv..

[12] Vijayakumar V., Ghosh M., Asokan K., Sukumaran S.B., Kurungot S., Mindemark J., Brandell D., Winter M., Nair J.R. (2023). 2D Layered Nanomaterials as Fillers in Polymer Composite Electrolytes for Lithium Batteries. Adv. Energy Mater..

[13] Yu X., Yang R., Zhang W., Yang X., Ma C., Sun K., Shen G., Lv F., Fan S. (2024). Interface engineering of polymer composite films for high-temperature capacitive energy storage. Chem. Eng. J..

[14] Kohli S., Rathee G., Jha I., Phor L., Sable H., Chaudhary V. (2025). Strategically engineering 2D MXene-based advanced adsorbents for sustainable wastewater remediation of dyes. Nanoscale.

[15] Zahid A., Mazhar H., Mujtaba G., Al-Mahmodi A.F., Qudoos A., Bangash I.A., Karim M.A., Khan H., Ali S.A. (2026). Biomass-modified nanoparticles for photocatalytic dye degradation: A sustainable roadmap toward a cleaner Future–Review. Biomass Bioenergy.

[16] Cui Y., Tibbitt M.W., Lu T.K., Tang T.-C. (2026). Design principles for adaptive and evolving engineered living materials. Curr. Opin. Biotechnol..

[17] Sasikumar K., Rajamanikandan R., Ju H. (2023). Nitrogen- and Sulfur-Codoped Strong Green Fluorescent Carbon Dots for the Highly Specific Quantification of Quercetin in Food Samples. Materials.

[18] Shameem A.S., Murugan A., Siva V., Palanisamy G., Kim I., Lee J., Paramasivam S. (2024). A High-Performance Supercapacitor Based on Hierarchical Template-Free Ni/SnO_2_ Nanostructures via Hydrothermal Method. Materials.

[19] Chen X., Cheng W., Liu H., Chen H., Ma J., Zhang Y., Wu Z., Wang C., You Y., Xing X. (2025). Research Progresses on Nano-Structured Silicon-Based Materials as Anode for Lithium-Ion Batteries. Materials.

[20] Xu J., Shen L., You H., Liu Y. (2024). An Aminobenzenethiol-Functionalized Gold Nanocolorimetric Sensor for Formaldehyde Detection. Materials.

[21] Zewude D.A., Akamatsu M., Ifuku S. (2024). Structural Color of Partially Deacetylated Chitin Nanowhisker Film Inspired by Jewel Beetle. Materials.

